# Malignant prediction of incidental findings using ring-type dedicated breast positron emission tomography

**DOI:** 10.1038/s41598-022-05166-2

**Published:** 2022-01-21

**Authors:** Shinsuke Sasada, Norio Masumoto, Akiko Emi, Takayuki Kadoya, Morihito Okada

**Affiliations:** Department of Surgical Oncology, Research Institute for Radiation Biology and Medicine, Hiroshima University, 1-2-3 Kasumi, Minami-Ku, Hiroshima City, Hiroshima 734-8551 Japan

**Keywords:** Breast cancer, Cancer imaging

## Abstract

The classification according to uptake patterns and metabolic parameters on ring-type dedicated breast positron emission tomography (dbPET) is useful for detecting breast cancer. This study investigated the performance of dbPET for incidental findings that were not detected by mammography and ultrasonography. In 1,076 patients with breast cancer who underwent dbPET, 276 findings were incidentally diagnosed before treatment. Each finding was categorized as focus (uptake size ≤ 5 mm), mass (> 5 mm), or non-mass (multiple uptake) according to uptake patterns. Non-mass uptakes were additionally classified based on their distributions as—linear, focal, segmental, regional, or diffuse. Thirty-two findings (11.6%) were malignant and 244 (88.4%) were benign. Visually, 227 (82.3%) findings were foci, 7 (2.5%) were masses, and 42 (15.2%) were non-masses. Malignant rates of focus, mass, and non-mass were 9.7%, 28.6%, and 19.0%, respectively. In the non-mass findings, 23 were regional and diffuse distributions, and presented as benign lesions. Focus uptake with low lesion-to-background ratio (LBR) and no hereditary risk were relatively low (2.7%) in breast cancer. In multivariate analysis, LBR and hereditary risk were significantly associated with breast cancer (*p* = 0.006 and *p* = 0.013, respectively). Uptake patterns, LBR, and hereditary risk are useful for predicting breast cancer risk in incidental dbPET findings.

## Introduction

The ^18^F-fluorodeoxyglucose positron emission tomography/computed tomography (FDG PET/CT) involves molecular imaging of glucose metabolism that is used for cancer screening, staging, monitoring treatment response, and predicting prognosis in breast cancer medicine^1–5^. Whole-body PET/CT cannot be used to visualize subcentimetric tumors because of its limited spatial resolution. Dedicated breast PET (dbPET) is a high-resolution molecular breast imaging technique with a high sensitivity for small breast cancers^6,7^. This advantage helps in visualizing the tumor heterogeneity and microenvironment, and evaluating residual tumor after treatment^8–10^. Additionally, dbPET helps in screening because the prognosis of subcentimetric breast cancers is favorable. However, no diagnostic classifications exist for dbPET, and the abnormal findings remain to be categorized. We have previously reported the usefulness of the classification of dbPET findings using uptake patterns and lesion-to-background ratio (LBR) for identifying malignancies^11^. However, the study was performed with breast cancer cases that were previously diagnosed, and thus, could not be generalized for breast cancer screening.

DbPET frequently predicts abnormal uptake that is not detected by other modalities^11,12^. Of these incidental findings, 10.5–32.5% were breast cancer. It is important to extract malignant lesions from the findings. However, there are no guidelines to distinguish between benign and malignant uptakes for incidental dbPET findings. The American College of Radiology (ACR) breast imaging-reporting and data system (BI-RADS) category 3 represents the malignant frequency of 0%–2%, and recommends that the findings be confirmed at short-interval follow-ups^13^. However, the cancer yield of category 3 findings on magnetic resonance imaging (MRI) is higher than the ceiling rate of 2% recommended by the ACR^14^. Moreover, it remains unclear whether all incidental findings on dbPET should be examined histologically or with short-interval follow-up. Previous studies have suggested that uptake patterns and LBR are related to breast cancer^11,12^. Here, we investigated whether the classification according to uptake patterns and metabolic features is also useful for incidental findings on dbPET, including hereditary risk, in breast cancer.

## Results

### Baseline characteristics

Of the 1520 abnormal findings, 276 (18.2%) were incidentally identified. Uptake patterns of these findings were as follows: 227 (82.3%) foci, 7 (2.5%) masses, and 42 (15.2%) non-masses. The non-mass findings included 1 linear, 11 focal, 7 segmental, 14 regional, and 9 diffuse distributions (Table 1). The median SUVmax and LBR were 3.0 and 1.5, respectively. Ninety-eight (35.5%) patients had an HBOC risk.

**Table 1:** Characteristics of incidental findings on dedicated breast PET.

	Number (%)
Uptake pattern	
Focus	227 (82.3)
Mass	7 (2.5)
Non-mass	42 (15.2)
Linear	1 (0.4)
Focal	11 (4.0)
Segmental	7 (2.5)
Regional	14 (5.1)
Diffuse	9 (3.3)
SUVmax (IQR)	3.0 (2.3–3.9)
Background SUV (IQR)	1.9 (1.6–2.4)
LBR (IQR)	1.5 (1.2–1.9)
Diagnosis	
Malignant	32 (11.6)
Benign	244 (88.4)
Pathological diagnosis	101 (36.6)
Radiological diagnosis	143 (51.8)
HBOC risk	98 (35.5)

HBOC, hereditary breast and ovarian cancer; IQR, interquartile range; LBR, lesion-to-background ratio; SUVmax, maximum standardized uptake value.

### Probabilities of malignant lesions

**Figure 1:**
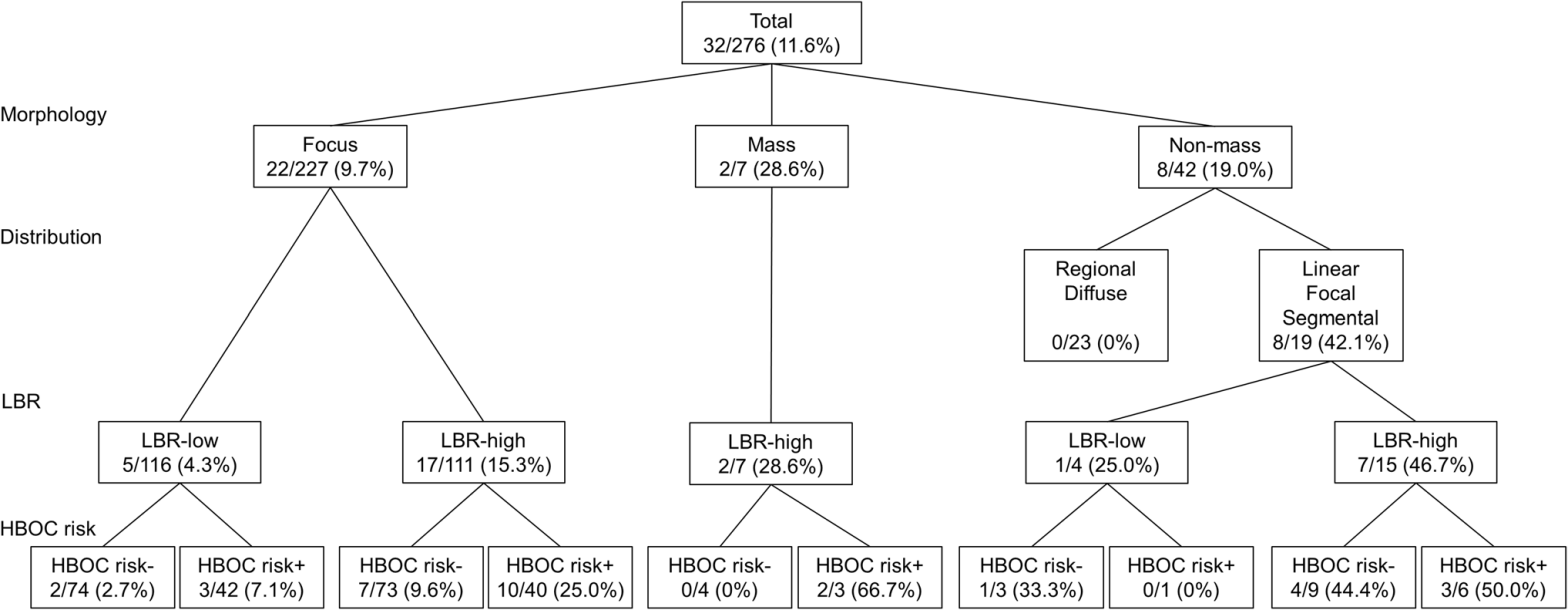
Probabilities of malignant lesions according to uptake patterns, LBR, and hereditary risk in incidental uptake on dedicated breast PET. HBOC risk is defined as having either contralateral breast cancer or family history of breast and ovarian cancers. *PET* positron emission tomography, *HBOC* hereditary breast and ovarian cancer, *LBR* lesion-to-background ratio.

Of the incidental findings, 32 (11.6%) were histologically confirmed as breast cancer. One hundred and one (36.6%) lesions were pathologically confirmed as benign, and 143 (51.8%) were radiologically evaluated as BI-RADS category ≤ 2, even after a median follow-up of 40 months. The probabilities of malignant lesions are shown in Fig. 1, based on uptake patterns, LBR, and HBOC risk. The malignant rates were as follows: 9.7% (22/227) in foci, 28.6% (2/7) in masses, and 19.0% (8/42) in non-masses. In the non-mass findings, following number of lesions were malignant: 0% (0/23) of regional and diffuse distributions, 100% (1/1) of linear, 18.2% (2/11) of focal, and 71.4% (5/7) of segmental distributions (Table 2). The area under the curve of LBR for predicting malignancies was 0.68 (95% confidence interval [CI], 0.58–0.77), and cutoff value was 1.437. The malignant lesions consisted of 13 invasive breast carcinomas (11 infiltrating duct carcinomas not otherwise specified, 1 tubular carcinoma, and 1 apocrine adenocarcinoma) and 19 ductal carcinomas in situ. The median size of invasive breast carcinomas was 3 mm (range: 0.3–12), and the median diameter of ductal carcinomas in situ was 7 mm (2–55). Among 32 incidental breast cancers, 10 were found in the contralateral breast, and they underwent simultaneous bilateral surgery. The benign lesions included 77 mastopathies, 17 papillomas, 12 fibroadenomas, 5 ductal adenomas, and 4 others.

**Table 2:** Frequency of malignancy according to uptake patterns and LBR on dedicated breast PET.

	Benign (n = 244)	Malignant (n = 32)	*P*
Uptake pattern			< 0.001
Focus	205 (90.3)	22 (9.7)	
Mass	5 (71.4)	2 (28.6)	
Non-mass	34 (81.0)	8 (19.0)	
Distribution			< 0.001
Linear	0 (0)	1 (100)	
Focal	9 (81.8)	2 (18.2)	
Segmental	2 (28.6)	5 (71.4)	
Regional	14 (0)	0 (0)	
Diffuse	9 (100)	0 (0)	
LBR			< 0.001
Low	122 (95.3)	6 (4.7)	
High	122 (82.4)	26 (17.6)	

IQR, interquartile range; LBR, lesion-to-background ratio.

### Predictors for malignant lesions

In multivariate analysis, linear, focal and segmental distributions of non-mass findings, high LBR, and HBOC risk were independent predictors of breast cancer (odds ratio [OR] 5.54, *p* = 0.002; OR 3.84, *p* = 0.006; and OR 2.76, *p* = 0.013, respectively) (Table 3). The low-risk classifications for malignancies were non-mass uptake with regional and diffuse distributions (0%), followed by focus uptake with low LBR, and no HBOC risk (2.7%). Mass and non-mass findings with other distributions had a high malignant risk regardless of LBR.

**Table 3:** Logistic regression analysis for predicting malignancy.

Factors	Univariate analysis		Multivariate analysis	
	OR (95% CI)	*P*	OR (95% CI)	*P*
Mass (vs focus)	3.73 (0.68–20.4)	0.129	2.17 (0.37–12.6)	0.389
Non-mass_RD (vs focus)	0.00 (0.00-Inf)	0.990	0.00 (0.00-Inf)	0.990
Non–mass_LFS (vs focus)	6.78 (2.46–18.6)	< 0.001	5.54 (1.88–16.4)	0.002
High LBR	4.33 (1.72–10.9)	0.002	3.84 (1.47–10.1)	0.006
HBOC risk	2.64 (1.25–5.57)	0.011	2.76 (1.24–6.17)	0.013

CI, confidence interval; HBOC, hereditary breast and ovarian cancer; LBR, lesion-to-background ratio; LFS, linear, focal and segmental distributions; OR, odds ratio; RD, regional and diffuse distributions.

### Images

**Figure 2:**
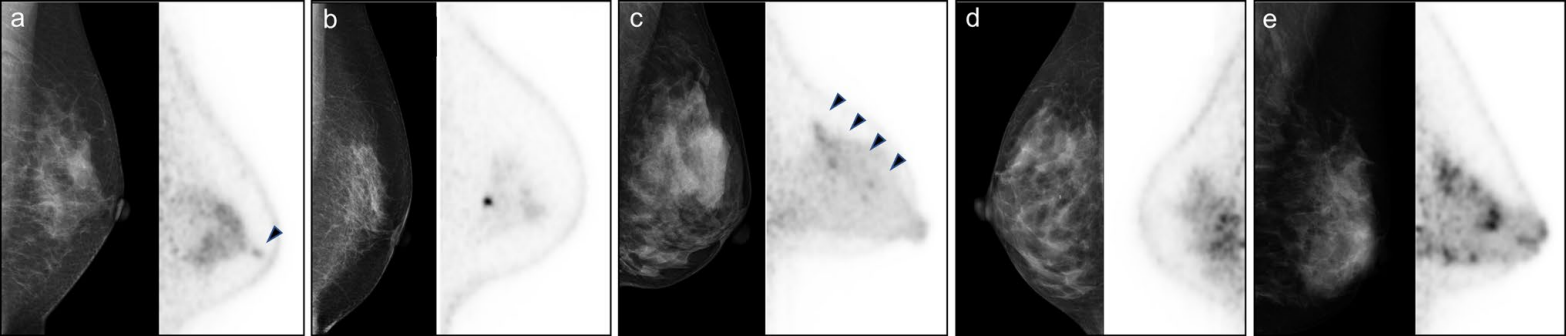
Representative images of incidental findings on dedicated breast PET and the diagnosis. (**a**) Focus uptake of 4 mm in diameter with lesion-to-background ratio (LBR) of 1.06 in the left breast (arrow head) and it is diagnosed with fibroadenoma. (**b**) Focus uptake of 4 mm in diameter with LBR of 3.19 in the left breast and it is diagnosed with infiltrating duct carcinoma not otherwise specified. (**c**) Non-mass uptake with segmental distribution of 50 mm in diameter in the left breast (arrow heads) and it is diagnosed with 1.5 mm of infiltrating duct carcinoma with 48 mm of intraductal spreading. (**d**) Non-mass uptake with regional distribution in the right breast and it is diagnosed with fibroadenoma. (**e**) Non-mass uptake with diffuse distribution in the left breast and it is diagnosed with mastopathy. Left, mammography; right, dedicated breast PET image; PET, positron emission tomography.

Representative images of mammography and dbPET are shown in Fig. 2. Focus uptakes were 4 mm in diameter, and the lesions were diagnosed with fibroadenoma and infiltrating duct carcinoma with LBR of 1.06 and 3.19, respectively (Fig. 2a,b). Non-mass uptake with segmental distribution was diagnosed as infiltrating duct carcinoma with an extensive intraductal component (Fig. 2c). Non-mass uptakes with regional and diffuse distributions were diagnosed as fibroadenoma and mastopathy, respectively (Fig. 2d,e).

## Discussion

This study investigated the malignant probability of incidental findings on ring-type dbPET in patients with breast cancer and factors related to malignancies. Uptake patterns, LBR, and hereditary risk were useful for predicting breast cancer.

Positron emission mammography (PEM) and ring-type dbPET, such as Mammi-PET and Elmammo, have a high sensitivity of 92–95% and 90.3–100%, respectively^6,15–17^. Ring-type dbPET has some advantages over PEM. First, ring-type dbPET provides three-dimensional images and makes it easy to identify the position of abnormal findings in the breast. This facilitates identification by a second-look US with US-guided biopsy and histological examination. Second, ring-type dbPET can calculate the SUV owing to attenuation and scatter correction^18^. Therefore, ring-type dbPET can help establish the precise classification of abnormal findings.

The dbPET is highly sensitive even for subcentimetric tumors because of its high resolution, with a sensitivity of 81.9%^6^. Therefore, dbPET can frequently detect unexpected uptakes, with a frequency of 7.6%^12^. Incidental findings were found in 17.7% of the patients in the present study. The difference in the frequency may be due to difference in purpose of the examination: cancer screening vs. preoperative detailed examination. Moreover, there was a large deviation in the percentages of mass uptake of 76.9% and 2.5% in the two settings. A majority of the incidental findings in the present study were foci (82.3%) and non-mass (15.2%) uptakes, because the preceding mammography and US detected large lesions. Thus, it is important to clarify the malignant probability of focus and non-mass findings. The malignant risk of focus uptake was stratified by LBR and HBOC risk, and that of non-mass was primarily determined by distribution patterns. Almost all classifications had a risk higher than 2%, which was the upper limit for BI-RADS category 3, excluding non-mass findings with regional and diffuse distributions^13^. Therefore, it is difficult to analyze dbPET findings using BI-RADS categories. An overestimation of low LBR focus uptake in patients without hereditary risk should be avoided. However, the significance of HBOC risk for mass and non-mass findings could not be assessed due to the small sample size.

In the present study, additional breast cancers were identified in 3.0% of the patients, and 2.0% were ipsilateral and 1.0% were contralateral breast cancers. Previous studies reported detection rates of 10.8% and 0.8% using PEM for additional breast cancer in the ipsilateral breasts and contralateral breasts, respectively^19,20^. In 580 women who underwent ring-type dbPET, 13 (2.2%) had additional breast cancers^12^. DbPET rarely detects additional cancers in patients who underwent conventional breast examinations.

This study has some limitations. First, it was a single-institution retrospective study. Second, all patients who had already been diagnosed with breast cancer underwent dbPET as a pre-treatment examination. Therefore, they have a high-risk potential for breast cancer, and the findings in this study cannot be directly extrapolated to the categorization for cancer screening. Third, tissues with abnormal uptake cannot be collected using dbPET. If US and MRI examinations cannot detect lesions, abnormal findings on dbPET are not histologically diagnosed. In the present study, approximately half of the findings were radiologically diagnosed as benign lesions, and they were confirmed to have no malignant findings after a median follow-up period of 40 months by US or enhanced MRI.

In conclusion, uptake patterns, LBR, and hereditary risk were useful for predicting breast cancer risk in incidental findings on dbPET. Therefore, incidental abnormal findings have a high probability of breast cancer and correspond to BI-RADS category ≥ 4. Non-mass uptakes with benign distributions, followed by low LBR focus without hereditary risk, have a low-risk of breast cancer. As the present study provides important implications for assessment of incidental findings on dbPET, it warrants further studies for categorization in cancer screening.

## Methods

### Patients

Of the patients diagnosed with breast cancer between January 2016 and December 2020 at our institute, 1,076 underwent ring-type dbPET before treatment. All patients underwent mammography and ultrasonography (US) before dbPET. Incidental findings were defined as those that were not noted on mammography or US. Hereditary risk was defined, according to hereditary breast and ovarian cancer (HBOC) risk, as history of contralateral breast cancer and family history of breast and ovarian cancers. This study was approved by the Ethical Committee for Epidemiology of Hiroshima University, and written informed consent was waived for this type of study (E-559). All procedures performed in studies involving human participants were in accordance with the ethical standards of the institutional research committee and with the 1964 Helsinki declaration and its later amendments or comparable ethical standards.

### DbPET examination

DbPET was performed using an Elmammo scanner (Shimadzu, Kyoto, Japan) in the prone position approximately 1.5 h after FDG injection (3–3.7 MBq/kg). The patients fasted for at least 4 h before the examination. The detector consisted of 36 detector modules arranged in three contiguous rings, four layers of 32 × 32 cerium-doped lutetium gadolinium oxyorthosilicate crystal array (crystal size: 1.44 × 1.44 × 18 mm), a light guide, and a 64-ch position-sensitive photomultiplier tube. The field of view was 185 × 156.5 mm, scan time was 7 min per bed position, and acquired data were reconstructed as 236 × 236 matrix images (pixel size, 0.78 × 0.78 mm) using a 3-dimensional dynamic row-action maximum likelihood algorithm.

DbPET image evaluation and quantification of the maximum standardized uptake value (SUVmax) were performed using Xeleris workstation (version 4.1; GE Healthcare). Volumes of interest (VOIs) with a diameter of 20 mm were delineated to include the entire abnormal uptakes on attenuation-corrected FDG PET images and within the ipsilateral normal breast tissue for the background uptake, and the SUVmax was calculated. If the accumulation range was larger than 20 mm, the whole lesion was measured by the multiple-shifted VOIs. The SUV display range was set at 0–7 as the workstation default. The attenuation correction of dbPET was performed as a homogeneous soft tissue of breast tissue composed of mammary glands and fat. LBR was defined as a ratio of the SUVmax of the lesion and SUVmax of background breast tissue. All PET images were evaluated independently by two radiologists with over 10 years of experience in nuclear medicine.

### Classification of dbPET findings

The classification of dbPET uptake patterns has been previously described^11^. Abnormal uptake on dbPET images was categorized as foci (uptake size ≤ 5 mm), masses (> 5 mm), or non-masses (multiple uptakes without distinct features of a mass) in reference to the BI-RADS MRI classification system^13^. Non-mass findings were further categorized by distribution as linear, focal, segmental, regional, or diffuse.

### Final diagnosis of dbPET findings

Breast findings identified by dbPET were evaluated pathologically or by additional radiological imaging. Lesions detected by a second-look US were pathologically confirmed by fine-needle aspiration or needle biopsy. All malignant lesions were histologically confirmed. Benign lesions were defined as histologically benign or BI-RADS category ≤ 2, and followed for at least 6 months (median 40 months) by US or enhanced MRI.

### Statistical analyses

The summarized data are presented as numbers and percentages, unless otherwise stated. Frequencies were compared using Fisher’s exact test for categorical variables. The receiver operating characteristic curve of the parameter was drawn to determine the cutoff value of the LBR. Logistic regression analysis was used to identify the predictors for malignant tumors. Statistical significance was set at *p* < 0.05. All statistical analyses were performed using EZR version 1.54 (Saitama Medical Center, Jichi Medical University, Saitama, Japan), a graphical user interface for R version 4.0.3 (The R Foundation for Statistical Computing, Vienna, Austria)^21^.
